# JMJD3 promotes survival of diffuse large B-cell lymphoma subtypes via distinct mechanisms

**DOI:** 10.18632/oncotarget.8836

**Published:** 2016-04-19

**Authors:** Yan Zhang, Long Shen, Dwayne G. Stupack, Nan Bai, Jing Xun, Guosheng Ren, Jihong Han, Luyuan Li, Yunping Luo, Rong Xiang, Xiaoyue Tan

**Affiliations:** ^1^ Department of Immunology, Medical School of Nankai University, Tianjin 300071, China; ^2^ Department of Pathology, Medical School of Nankai University, Tianjin 300071, China; ^3^ UCSD Moores Cancer Center, University of California, San Diego, La Jolla CA 92093, USA; ^4^ Molecular Oncology and Epigenetics Laboratory, The First Affiliated Hospital of Chongqing Medical University, Chongqing 400016, China; ^5^ The College of Life Sciences, Nankai University, Tianjin 300071, China; ^6^ Department of Immunology, Beijing Union Medical School, Beijing, 100010, China; ^7^ Central Laboratory, Logistics University of Chinese People's Armed Police Force, Tianjin, 300162, China

**Keywords:** DLBCL, apoptosis, JMJD3, IRF4, Bcl-2

## Abstract

JMJD3 (Jumonji domain containing-3), a histone H3 Lys27 (H3K27) demethylase, has been reported to be involved in the antigen-driven differentiation of germinal center B-cells. However, insight into the mechanism of JMJD3 in DLBCL (Diffuse large B-cell lymphoma) progression remains poorly understood. In this study, we investigated the subtype-specific JMJD3-dependent survival effects in DLBCL. Our data showed that in the ABC subtype, silencing-down of JMJD3 inhibited interferon regulatory factor 4 (IRF4) expression in a demethylase activity-dependent fashion. IRF4 reciprocally stimulated expression of JMJD3, forming a positive feedback loop that promoted survival in these cells. Accordingly, IRF4 expression was sufficient to rescue the pro-apoptotic effect of JMJD3 suppression in the ABC, but not in the GCB subtype. In contrast, ectopic overexpression of BCL-2 completely offset JMJD3-mediated survival in the GCB DLBCL cells. *In vivo*, treatment with siRNA to JMJD3 reduced tumor volume concordant with increased apoptosis in either subtype. This suggests it is a common target, though the distinctive signaling axes regulating DCBCL survival offer different strategic options for treating DLBCL subtypes.

## INTRODUCTION

Transcriptional repression and activation of critical genes, as regulated by post-translational modification of histone tails, plays an essential role in the developmental process or cellular differentiation. This includes neural differentiation, the lineage commitment of embryonic stem cells, and terminal differentiation of progenitor cells. One important repressive modification, histone methylation on specific lysine residues, is manipulated by different families of histone lysine methyltransferases and demethylases, including Polycomb group (PcG) of proteins, LSD1 and JmjC-domain-containing proteins [1]. The mutation or dysregulation of these proteins have been reported to be related to cancer and other diseases [2–5].

JMJD3 and UTX are demethylases that remove the tri-methyl mark from H3K27. They activate the transcriptional expression of genes by demethylating H3K27me3 and disassociating polycomb group proteins. JMJD3 expression is influenced by both specific gene expression and cellular context. This contributes to both differentiation [6–11] and tumor suppression [12–14], as well as tumor cell survival [15, 16]. It is notable that JMJD3 plays a role in the B-cell differentiation. Moreover, JMJD3 is aberrantly overexpressed and seems to contribute to pathogenesis of Epstein-Barr virus-associated Hodgkin's lymphoma [17–19].

Diffuse large B-cell lymphoma (DLBCL) is the most common type of Non-Hodgkin lymphoma. It is an aggressive and heterogeneous disease comprising two major distinct subtypes (ABC and GCB subtypes) that responds differently to standard treatments. Various genetic lesions, including mutations in PRDM1, CARD11, CD79B, TNFAIP3, MYD88 and translocations of MYC, BCL-2 and BCL6, have been shown to promote the tumorigenesis and the survival of DLBCL [20–28]. Furthermore, the majority of lesions targeting histone or chromatin modification are also a prominent feature of the DLBCL [19, 29–31]. In this study, we showed that aberrantly upregulated JMJD3 exerts an anti-apoptotic effect in DLBCL and acted as a potent positive regulator of DLBCL survival. Moreover, we characterized distinct mechanisms by which JMJD3 regulates survival in different subtypes of DLBCL.

## RESULTS

### JMJD3 is over-expressed in tumor tissues of DLBCL and cultured DLBCL cell lines

**Figure 1:**
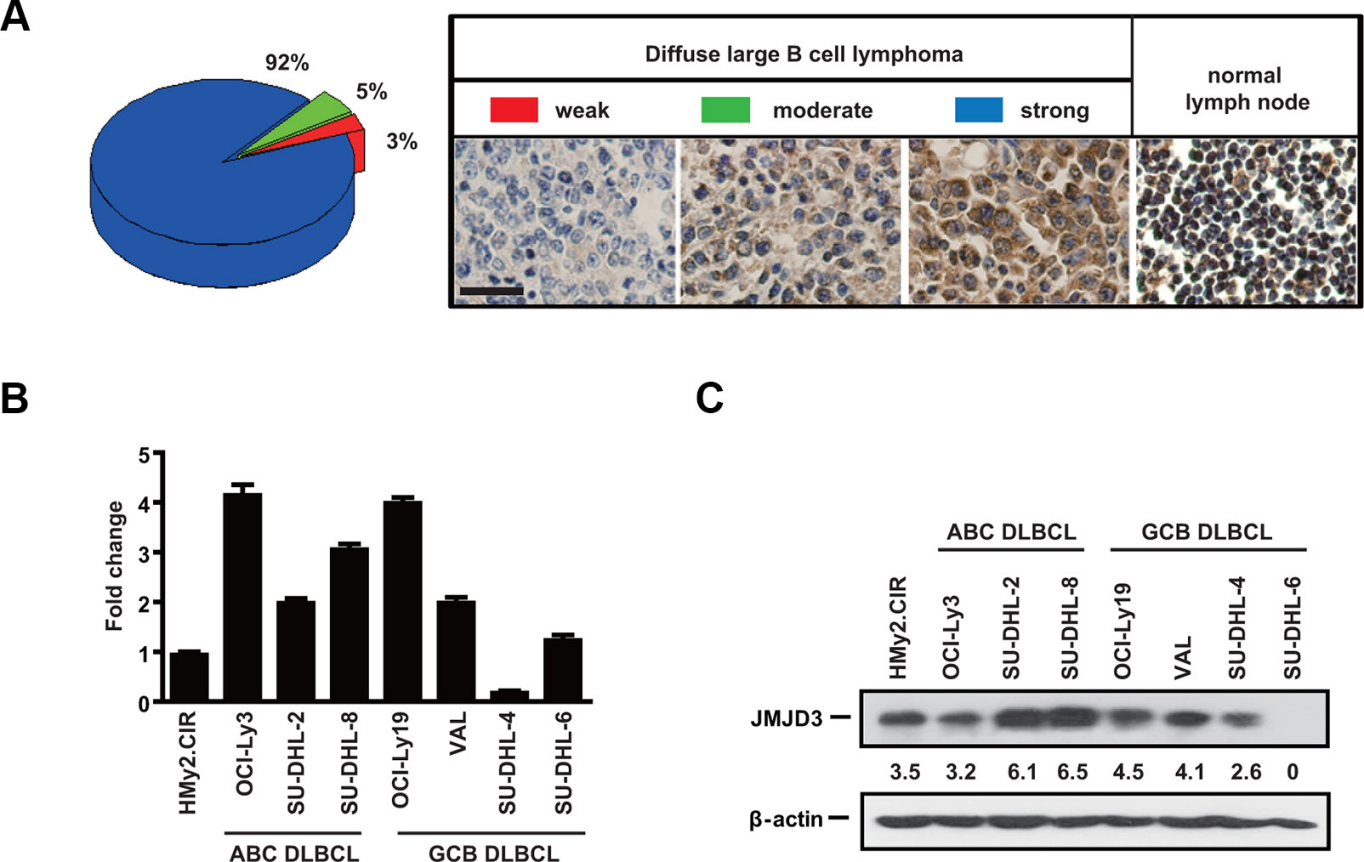
The expression of JMJD3 in DLBCL patients and cell lines is heterogeneous (**A**) The distribution of JMJD3 heterogeneous expression in DLBCL tissues samples from 100 patients (left), Immunohistochemistry showing the variant expression of JMJD3 in DLBCL tissues and normal lymph node (right). Scale bar, 50 μm. (**B**) RNA (shown by real time PCR) levels of JMJD3 in normal B cell line and DLBCL cell lines. GAPDH has been used as a loading control. (**C**) JMJD3 protein was resolved by immunoblotting and levels in the normal B cell and DLBCL cell lines were quantified via densitometry.

To investigate the expression level of JMJD3 in DLBCL, we first performed immunohistochemical analysis of JMJD3 on the tissue microarray composed of 100 surgical DLBCL samples. The intensity of immunostaining was scored using a scale from 0 to 3, and categorized the samples into three main groups, low (0 – 1), moderate (2) or high (3). Hierarchical clustering was performed on the data segregating the tumors into three groups based on the intensity of JMJD3 staining. Relative to normal lymph node controls, expression was strong in 92% of the DLBCL samples. Similarly, high levels of expression were also observed in Hodgkin's lymphoma, mucosa-associated B cell lymphoma and T cell lymphoma samples (data not shown) (Figure 1A). This suggested a general tumor-facilitating role for JMJD3. *In vitro*, although highly heterogeneous, JMJD3 expression was generally elevated in most DLBCL cell lines when compared with normal B cell line (HMy2.CIR) at both the mRNA and protein levels (Figure 1B and 1C).

### Depletion of JMJD3 induces apoptosis in DLBCL cells

**Figure 2:**
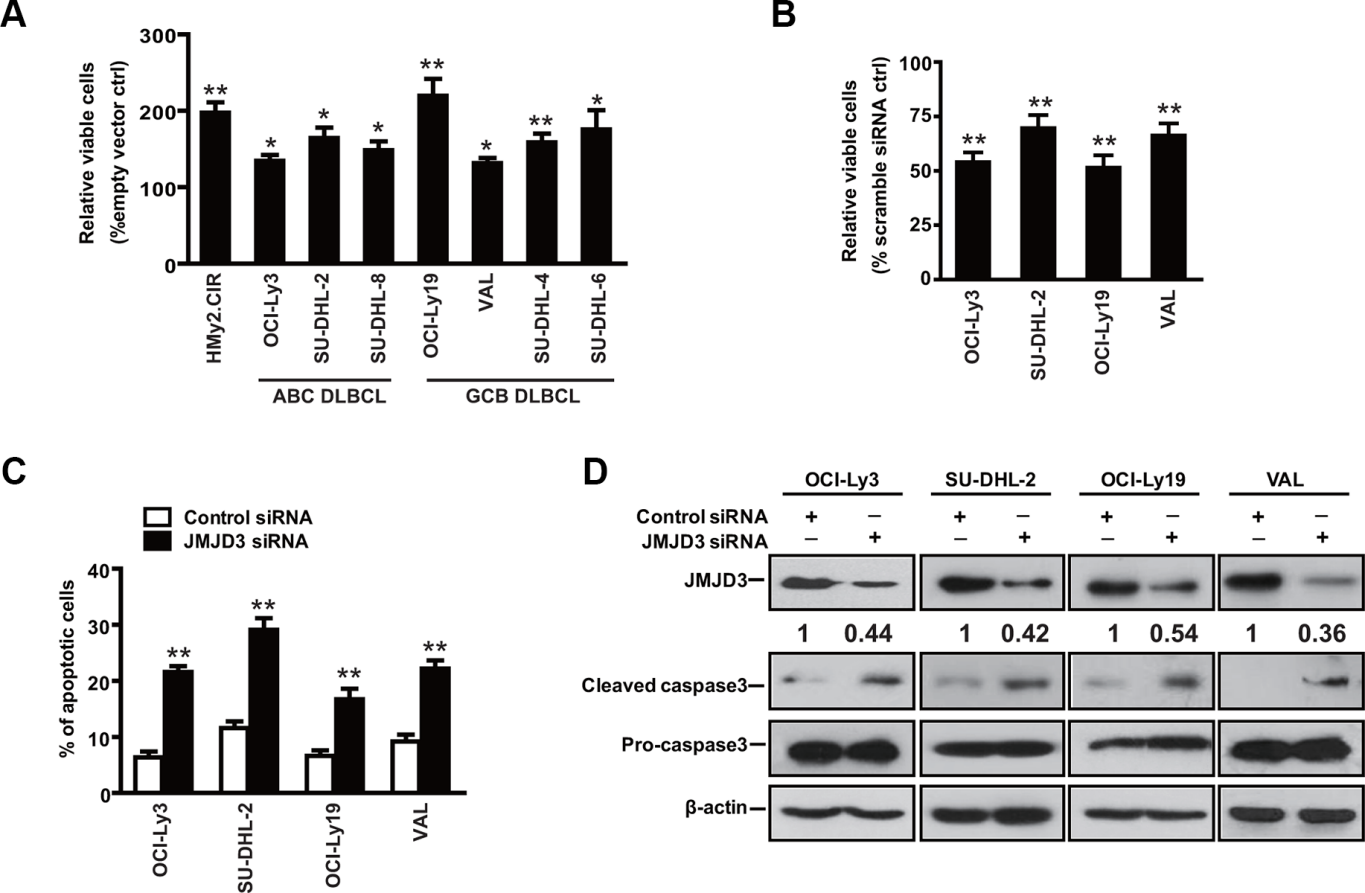
Downregulation of JMJD3 promotes apoptosis in DLBCL cell The viability of ABC and GCB DLBCL cells ectopically expressing JMJD3 (**A**) or with siRNA-suppressed JMJD3 expression (**B**) was evaluated after 3 days. (**C**) Representative apoptosis analysis using annexin-V/PI staining and flow cytometry of DLBCL cells treated with scrabble control siRNA or JMJD3 siRNA for 48 h. (**D**) Immunoblot assay showing the depletion of JMJD3 proteins in DLBCL cells as compared with the scrabble control siRNA and the effect of JMJD3 depletion on cleaved caspase3 and pro-caspase3 expression in DLBCL cells. The data shown are the mean ± S.E.M. of at least three independent experiments (*P < 0.05, **P < 0.01, Student's *t*-test).

To investigate the role of JMJD3 in the pathogenesis of DLBCL, we first assessed the effect of JMJD3 on the survival in the cultured cell line models of DLBCL. Ectopic over-expression of JMJD3 increased cellular viability in the control B cells and DLBCL cells, while suppression of JMJD3 decreased the viability of DLBCL cells (Figure 2A and 2B). Then, FACS analysis of annexin-V/propidium iodide (PI) stained samples were performed to evaluate the apoptotic level. Our data revealed that suppression of JMJD3 induced apoptosis in the DLBCL cells (Figure 2C). Consistent with a role for JMJD3 in preventing apoptosis, suppression of JMJD3 stimulated maturation of caspase-3 (Figure 2D). Taken together, these results suggest that JMJD3 plays an critical role in the regulation of apoptosis in DLBCL cells.

### IRF4 is a target gene regulated by JMJD3

**Figure 3:**
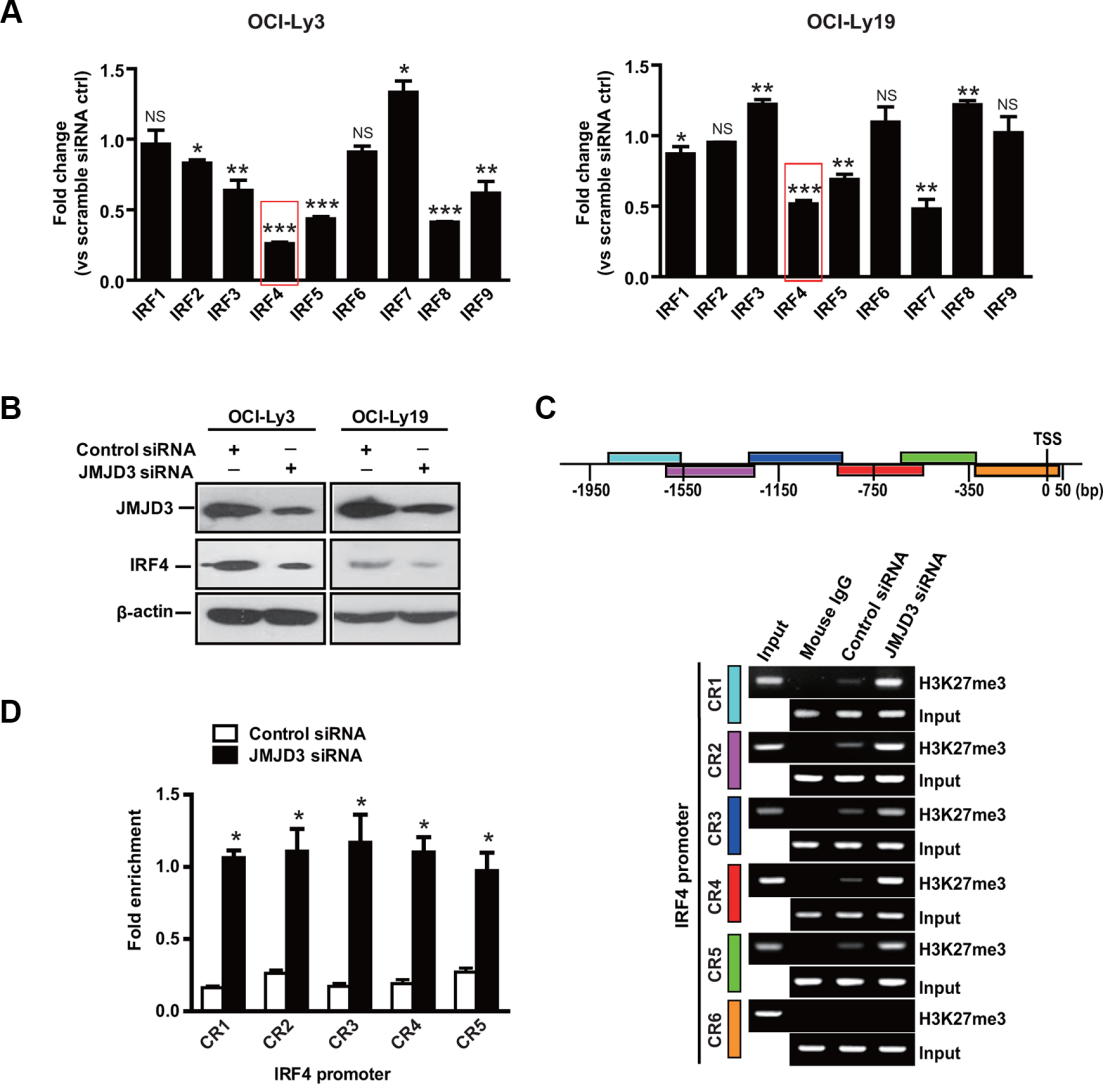
IRF4 is a downstream gene regulated by JMJD3 (**A**) Expression levels of genes of interferon regulatory factor family (IRF1, IRF2, IRF3, IRF4, IRF5, IRF6, IRF7, IRF8, and IRF9) in OCI-Ly3 (ABC DLBCL, left panel) and OCI-Ly19 (GCB DLBCL, right panel) cells depleted or not of JMJD3 via JMJD3 siRNA or scrambled control siRNA. The mRNA expression levels were determined by real time PCR and normalized against the expression levels of GAPDH. (**B**) Immunoblot assay evaluating JMJD3 depletion and IRF4 expression in OCI-Ly3 and OCI-Ly19 cells. (**C**) Schematic diagram of IRF4 promoter regions (upper panel). ChIP-PCR analysis of H3K27me3 mark of the IRF4 promoter in OCI-Ly3 cells depleted JMJD3 or not. Mouse IgG was used as a negative control, with input used as loading control (lower panel). (**D**) Quantification of the ChIP-PCR analysis results (quantification of densitometric measurement of signal intensity shown). The data shown are the mean ± S.E.M. of three independent experiments (*P < 0.01, Student's *t*-test).

Interferon regulatory factor (IRF) family plays central roles in the cellular differentiation of hematopoietic cells [32]. Moreover, several IRF family members exert important effects on the regulation of both cell cycle and apoptosis in DLBCL [33, 34]. Next, we characterized the effect of JMJD3 depletion on the regulation of 9 IRF family genes in DLBCL cells. Real-time PCR analysis revealed that depletion of JMJD3 by specific siRNA remarkably suppressed the expression of IRF4 and IRF5 in both ABC (OCI-Ly3) and GCB (OCI-Ly19) DLBCL cells (Figure 3A and 3B). Since IRF4 expression acts as a hallmark to dividing ABC and GCB DLBCL [35], we therefore subjected IRF4 expression to further analysis.

Chromatin immunoprecipitation (ChIP) assays were used to identify whether JMJD3 expression influenced H3K27 trimethylation in the IRF4 promoter region. As previously shown [11], a sharp increase in H3K27 trimethylation was observed at the IRF4 promoter after JMJD3 suppression (Figure 3C and 3D). Collectively, these results suggest that silencing-down of JMJD3 inhibits the expression of IRF4 via increase H3K27 trimethylation at the IRF4 promoter.

### NF-κB signaling mediates the JMJD3-IRF4 axis induced survival in ABC DLBCL cells

**Figure 4:**
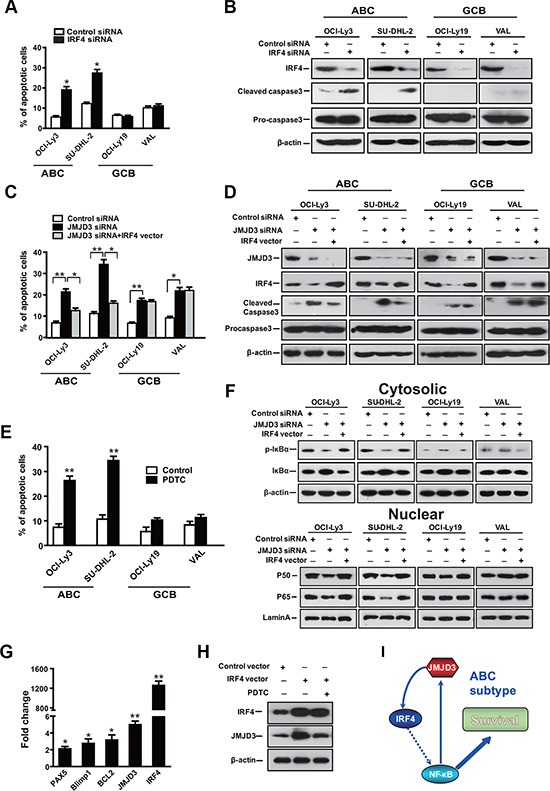
The JMJD3-IRF4 axis mediates the anti-apoptotic effect via NF-κB signal pathway in ABC DLBCL (**A**) Measurement of apoptosis using annexin-V/PI staining and flow cytometry of DLBCL cells following suppression of IRF4 via IRF4 siRNA, scrambled siRNA was used as control. (**B**) Immunoblot analysis showing the depletion of IRF4 protein in IRF4 siRNA group as compared with the scrambled control siRNA group, as well as the effect of IRF4 depletion on caspase3 maturation. (**C**) Measurement of apoptosis using annexin-V/PI staining and flow cytometry of DLBCL cells that overexpression IRF4 following the depletion of JMJD3. (**D**) Immunoblot analysis evaluating the depletion of JMJD3 protein and overexpression of IRF4 protein in DLBCL cells and the combined effects of JMJD3 depletion and IRF4 overexpression on the expression of cleaved caspase3 and pro-caspase3 in DLBCL cells. (**E**) The effect of co-culture with an NFkB pathway inhibitor (PDTC) on ABC and GBC cell viability. (**F**) Immunoblot assay showing the combined effects of JMJD3 depletion and IRF4 overexpression on p-I-κB and I-κB expression in the cytoplasm of DLBCL cells and on p50 and p65 expression in the nucleus of DLBCL cells relative to b-actin and lamin A, respectively. (**G**) Alterations in mRNA expression as evaluated by qPCR following ectopic expression of IRF4 in OCI-Ly3 cells. (**H**) Expression of IRF-4 and JMJD3 following ectopically overexpression of IRF-4 with or without PDTC. (**I**) Model for positive feedback along the JMJD3-IRF4-NFkB axis. The apoptosis and mRNA inductions shown are the mean ± S.E.M. of three independent experiments (*P < 0.01, **P < 0.001, Student's *t*-test).

To establish whether IRF4 is the downstream gene which mediates the apoptotic program triggered following JMJD3 depletion in DLBCL cells, we depleted IRF4 in both ABC (OCI-ly3 and SU-DHL-2) and GCB (OCI-ly19 and VAL) DLBCL cells. The depletion of IRF4 was sufficient to induce apoptosis in ABC, but not GCB subtypes of DLBCL (Figure 4A). Supporting these results, down-regulation of IRF4 only selectively induced the maturation of procaspase 3 in ABC cells (Figure 4B). Complementing these results, increased IRF4 expression only rescues JMJD3 depletion mediated apoptosis in the ABC subtype cells (Figure 4C and 4D). This data suggests that JMJD3 might exert anti-apoptotic effect via distinct regulatory mechanism in these two subtypes of DLBCL.

NF-κB pathway is constitutively activated in ABC subtype of DLBCL and IRF4 regulates the NF-κB gene expression signature [34]. Then, we determined the role of IRF4 in the pathogenesis of DLBCL. We tested whether IRF4 rescued the JMJD3 depletion induced apoptosis via suppressing NF-κB signaling. Inhibitor of NF-κB signaling selectively induced apoptosis in ABC cells (Figure 4E). We assessed the phosphorylation of IκBα, in the cytosol. In tandem, we also evaluated the nuclear translocation of NF-κB. As would be expected in this model, depletion of JMJD3 reduced IKK activity and the nuclear translocation of NF-κB in ABC DLBCL cells. Ectopic over-expression of IRF4 in JMJD3-depleted ABC cells was sufficient to rescue the inhibited NF-κB signaling (Figure 4F), suggesting that IRF4 mediates the anti-apoptotic effect of JMJD3 via NF-κB signal pathway in ABC cells. Interestingly, ectopic over-expression of IRF4 also up-regulated the expression of JMJD3 in an NFκB dependent manner (Figure 4G and 4H). Therefore, it suggested a positive feedback mechanism perpetuated this pro-survival pathway in ABC cells.

### BCL-2 mediates the survival effect of JMJD3 in GCB DLBCL cells

**Figure 5:**
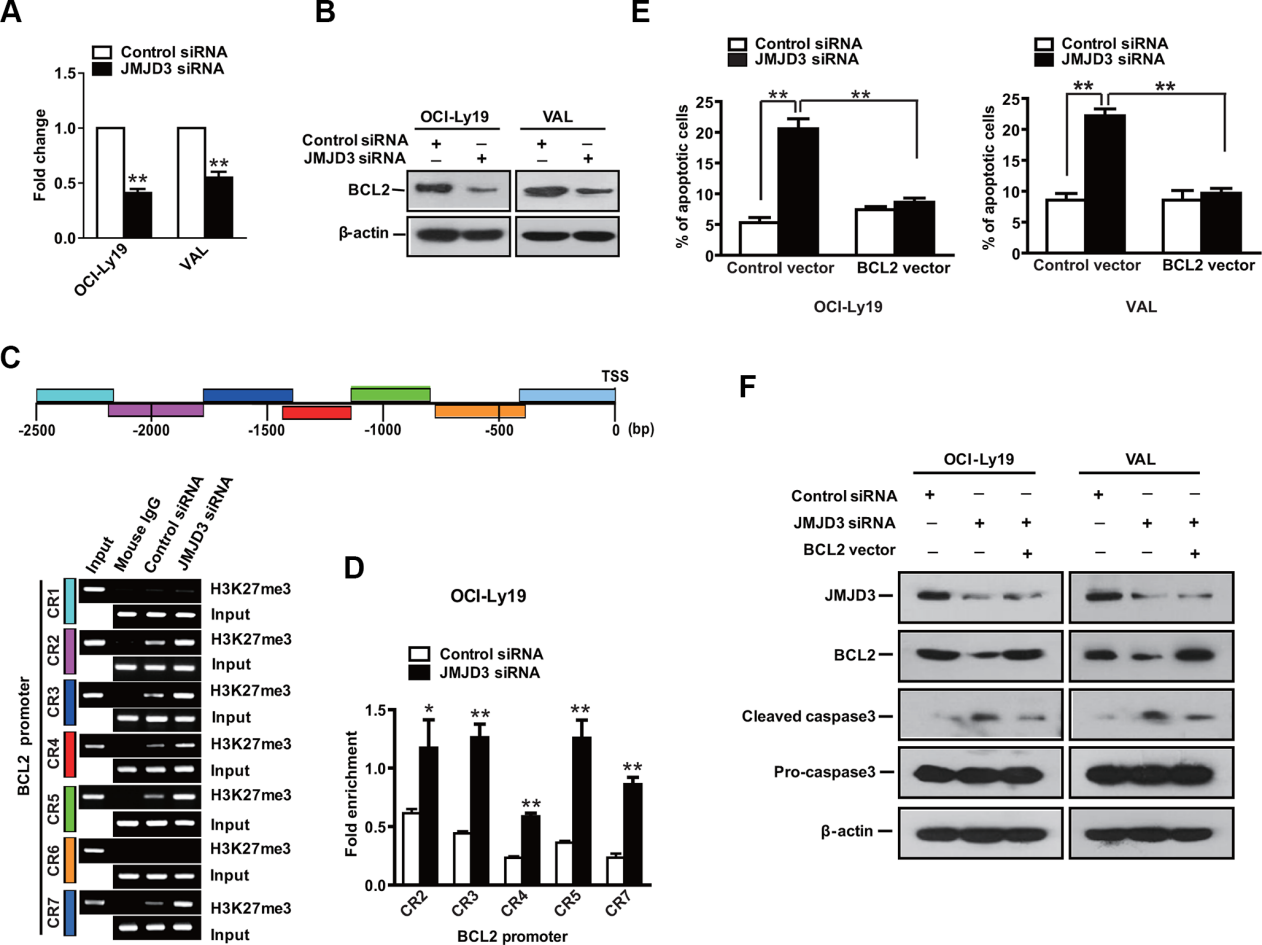
BCL-2 accounts for a significant survival component of JMJD3 in GCB DLBCL Bcl-2 mRNA as evaluated by qPCR (**A**) and Bcl2 protein (**B**) as resolved by immunoblotting using GCB DLBCL cells (OCI-Ly19 and VAL) following depletion of JMJD3 using specific siRNA, scramble siRNA used as control. (**C**) Schematic diagram of BCL-2 promoter regions (upper panel). ChIP-PCR analysis of H3K27me3 mark of the BCL2 promoter in OCI-Ly19 cells with depleted JMJD3 or control. (**D**) Densitometric quantification of the ChIP-PCR analysis results. (**E**) Measurement of apoptosis using annexin-V/PI staining and flow cytometry of OCI-Ly19 and VAL cells depleted of JMJD3 combined with expression of Bcl-2 or control. (**F**) Immunoblot assay showing the depletion of JMJD3 proteins and overexpression of Bcl-2 proteins in DLBCL cells and the combined effects of JMJD3 depletion and Bcl-2 overexpression on caspase3 maturation in GCB DLBCL cells. The data shown are the mean ± S.E.M. of three independent experiments (*P < 0.05, **P < 0.01, Student's *t*-test).

The effect that IRF4 rescued the apoptosis induced by JMJD3 depletion could only be observed in the ABC DLBCL cells, implicating alternative mechanism which mediates the JMJD3 related survival in the GCB DLBCL cells. In hormone-dependent breast cancer, the transcriptional activation of the survival gene *BCL2* is dependent on the simultaneous inactivation of H3K27 methyltransferase and subsequent demethylation of H3K27 at a poised enhancer by ERα-dependent recruitment of JMJD3 [16]. Therefore, we evaluated whether *BCL2* is a target gene of JMJD3 in GCB DLBCL cells. As shown (Figure 5A and 5B), a significant decrease in Bcl- 2 protein was observed upon depletion of JMJD3 in the GCB DLBCL cells. ChIP assays revealed an increase in H3K27 trimethylation at the *BCL2* promoter following depletion of JMJD3 in OCI-Ly19 cells (Figure 5C and 5D). Collectively, these results suggested that JMJD3 promoted the expression of Bcl-2 via suppressing the H3K27 trimethylation of *BCL2* promoter in GCB DLBCL cells. Accordingly, ectopic over-expression of *BCL2* was sufficient to attenuate apoptosis induced by JMJD3 depletion in the GCB DLBCL cells (Figure 5E and 5F). This data suggested that Bcl-2 mediates the cell-survival effect of JMJD3 among GCB DLBCL cells.

### Bcl-2 mediates a limited survival effect of JMJD3 in ABC DLBCL cells

**Figure 6:**
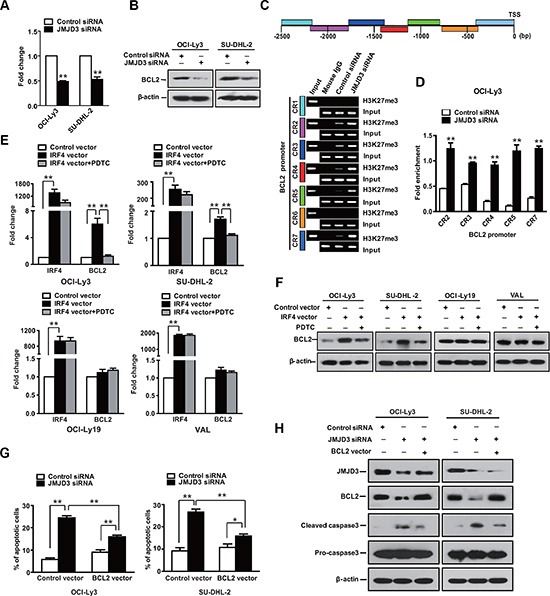
Bcl-2 has limited impact in ABC DLBCL The RNA (**A**) (qPCR) and protein (**B**) (immunoblot) expression levels of BCL-2 in OCI-Ly3 and SU-DHL-2 cells, two ABC DLBCL cell lines with depleted JMJD3 using specific siRNA or control siRNA. (**C**) Schematic diagram of *BCL2* promoter regions (upper panel). ChIP-PCR analysis of H3K27me3 mark of the *BCL2* promoter in OCI-Ly3 cells depleted JMJD3 or not. Mouse IgG used as negative control, input used as loading control (lower panel). (**D**) Quantification of the ChIP-PCR analysis results. Relative mRNA (**E**) and protein expression levels (**F**) of IRF4 and Bcl-2 in DLBCL cells overexpressing IRF4 or not, and in absence or presence of PDTC. (**G**) Measurement of cell death using annexin-V/PI staining and flow cytometry of ABC DLBCL cells OCI-Ly3 and SU-DHL-2 that overexpress Bcl-2 following the depletion of JMJD3. (**H**) Immunoblot assay showing the depletion of JMJD3 and overexpression of BCL2 in DLBCL cells, as well as the combined effects of JMJD3 depletion and Bcl-2 overexpression on cleaved caspase3 and pro-caspase3 expression in ABC DLBCL cells. The data shown are the mean ± S.E.M. of three independent experiments (*P < 0.05, **P < 0.01, Student's *t*-test).

Considering that Bcl-2 acts as a broad and potent cell survival effector, which is also expressed in the ABC subtype; therefore, we examined whether Bcl-2 expression was involved in the survival of the ABC DLBCL cells. Data showed that expression of Bcl-2 was decreased upon depletion of JMJD3 in the ABC DLBCL cells. ChIP assays revealed an increase in H3K27 trimethylation at the *BCL2* promoter following depletion of JMJD3 in OCI-Ly3 cells (Figure 6A–6D). These results suggested *BCL2* gene as target of JMJD3 in the ABC DLBCL cells. Notably, IRF4 stimulated the expression of Bcl- 2 and administration of NF-kB inhibitor blocked the IRF-induced Bcl-2 in the ABC cells (Figure 6E and 6F). However, ectopic Bcl-2 expression was not sufficient for complete rescue of ABC cells following the suppression of JMJD3, and maturation of procaspase-3 was still observed (Figure 6G and 6H). Thus, though Bcl-2 may also contribute to ABC cell survival, it is principally dependent upon NFkB signaling downstream of IRF4.

### Therapeutic delivery of JMJD3 siRNA inhibits DLBCL tumor growth and induces tumor cell death in a mouse model of disease

**Figure 7:**
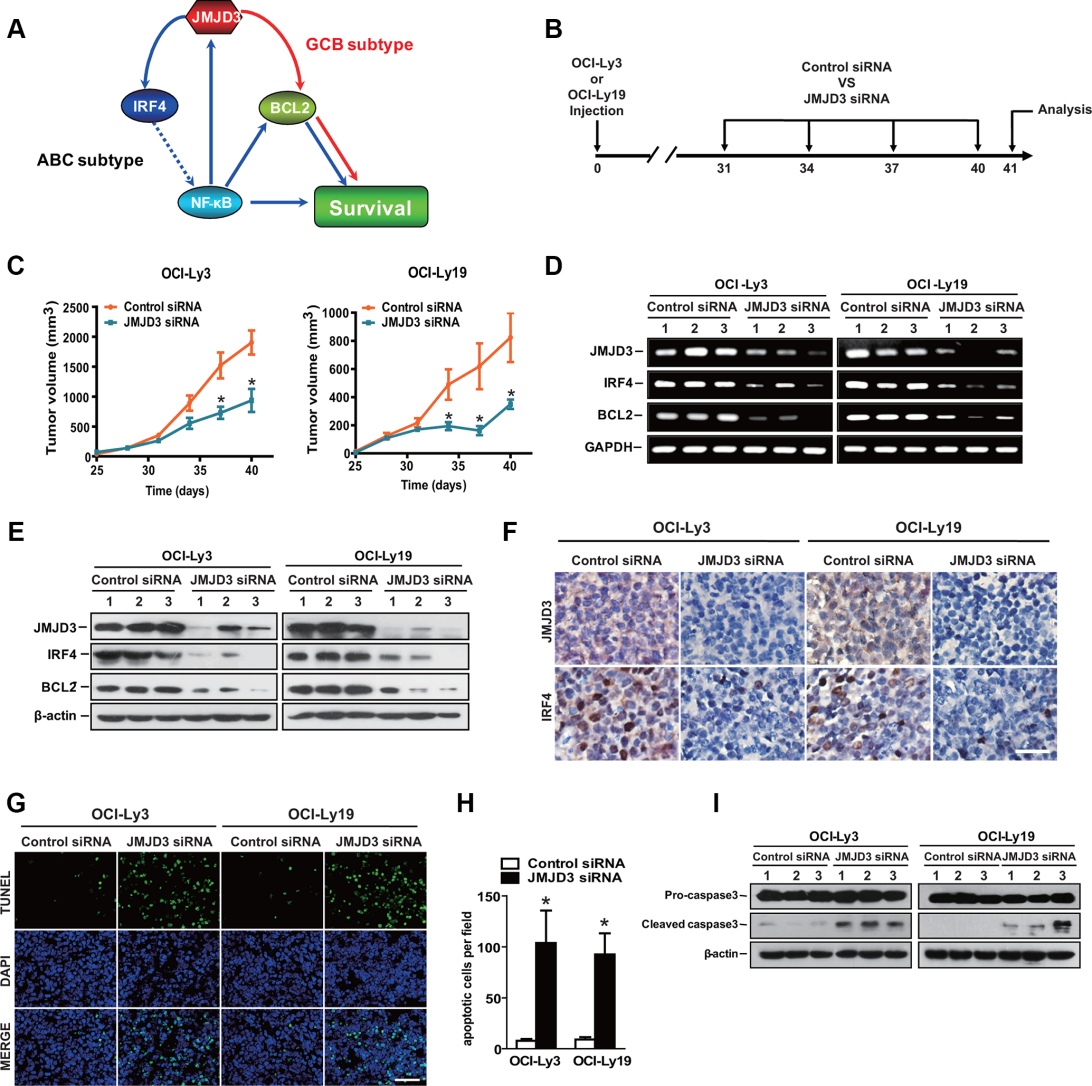
Therapeutic treatment with JMJD3 siRNA inhibits tumor growth and induces tumor apoptosis in DLBCL mouse model (**A**) Model of distinct mechanisms of JMJD3 action in DLBCL. (**B**) Mouse model and schedule of long-term treatments. The DLBCL tumor model was developed by subcutaneous injection of 1 × 10^7^ OCI-Ly3 (ABC DLBC) or OCI-Ly19 (GCB DLBC) cells into NOD/SCID mice on day 0. On day 31, 34, 37 and 40, the animals received injection of control siRNA or JMJD3 siRNA into tumors. All the tumors were isolated on day 41 for further analysis. (**C**) The tumor volume of OCI-Ly3 (left) and OCI-Ly19 (right) DLBCL tumor mouse models treated with JMJD3 siRNA or control siRNA. The RNA (**D**) (qPCR) and protein (**E**) (immunoblot) expression levels of JMJD3, IRF4 and BCL-2 in OCI-Ly3 and OCI-Ly19 established tumors treated with JMJD3 siRNA or control siRNA. (**F**) Immunohistochemical staining showing the expression and distribution of JMJD3 and IRF4 in OCI-Ly3 and OCI-Ly19 established tumors depleted or not of JMJD3. Scale bar, 50 μm. Representative pictures (**G**) and the quantification of the percentage of apoptotic tumor cells of TUNEL staining (**H**) in OCI-Ly3 and OCI-Ly19 established tumor sections. Scale bar, 50 μm. (**I**) Western blot assay showing the effect of JMJD3 depletion on pro-caspase3 and cleaved caspase3 in OCI-Ly3 and OCI-Ly19 DLBCL tumors (β-actin used as loading control). (*P < 0.05, Student's *t*-test).

Although JMJD3 played a key role in tumor survival for both types *in vitro* (As summarized in Figure 7A), it remained unclear whether this was recapitulated *in vivo*. We then evaluated the effect of silencing-down of JMJD3 on tumor progression in the mice with established DLBCL tumors. Mice were treated 4 times with intratumoral injections at 48 h intervals with either scrambled control siRNA, or JMJD3 siRNA at a final dose of 10 nmol per mouse (Figure 7B). Relative to the mice treated with scrambled controls, the JMJD3 siRNA treated group exhibited an arrested tumor growth (Figure 7C). Importantly, the expression of JMJD3, IRF4 and BCL2 in tumor tissues derived from JMJD3 siRNA-treated mice were dramatically decreased when compared to tumors from the control animals (Figure 7D–7F). TUNEL analysis revealed an increase in percentage of apoptotic cells in JMJD3-depleted tumors (Figure 7G and 7H), concordant with the increased maturation of procaspase-3 in the JMJD3 siRNA treated group (Figure 7I).

## DISCUSSION

Our current understanding of DLBCL delineates two major disease subgroups (ABC& GCB) that most likely reflect tumor derivation from B cells at discrete stages of differentiation. These two subtypes are associated with distinct genetic lesions, raising the notion that distinct mechanisms may regulate their survival [21–23, 27, 35]. Thus, while it was perhaps somewhat surprising to find that JMJD3 was critical to the survival of both subtypes, the precise mechanisms downstream of JMJD3 expression are somewhat different.

A hallmark of ABC DLBCLs is the constitutive activation of the nuclear factor-κB (NF-κB) pathway, which plays a pivotal role in the survival of ABC DLBCL. Therefore, it was not unexpected that NF-κB played an obligatory role in tumor survival. In this case, JMJD3 maintains cell survival principally by increasing IRF4 expression and amplifying NF-κB signaling, though some effect might be attributed to increased levels of Bcl-2. By contrast, among GCB cells, the chromosomal translocations that are observed (almost exclusively) are those involving the *BCL2* and *MYC* oncogenes. In fact, 30–40% of GCB DLBCL cases harbor t (14;18) (q32;q21), which results in deregulated expression of Bcl-2 [24, 36]. Since the *MYC* pathway promotes apoptosis via the intrinsic *BCL2*- dependent pathway [37], it is reasonable that the regulation of this survival pathway is both necessary and sufficient for the GCB tumor survival. Therefore, the suppression of *BCL2* expression by JMJD3 potentiates its amplification. It is interesting to speculate that in the minority of cases of GCB in which JMJD3 is not found to be elevated, there may be alternative mechanisms to enhance and/or sustain *BCL2* promoter activity.

The epigenetic amplification of IRF4 by JMJD3 is not likely to be unique to DLBCL. We observed elevated levels of the JMJD3 protein in other types of hematopoietic neoplasia, including both T and B cells lesions, suggesting that it may be a general mechanism to promote survival in the hematopoietic compartment, and supporting the idea that JMJD3 amplification is likely to be clinically important. Although JMJD3-IRF4 axis seems no direct effect on GCB, we cannot rule out the possibility that these cells may be more sensitive to apoptosis when directly challenged with a therapeutic agent or other pro-apoptotic insults. Saito et al. reported that relative low level of IRF4 in the GCB subtype blocked the downregulation of BCL6 expression, which is required by the post-germinal center B cell development [38]. Up till now, there is few data about the role of IRF4 in the GCB subtype of DLBCL. It has been reported that IRF4 inhibits cell cycle progression of GCB-derived Burkitt's lymphoma cells and induces terminal differentiation toward plasma cells through mechanisms independent of BCL6 downregulation [39]. In addition, the activation and polarization of macrophages in response to bacterial or helminth infection show that the JMJD3-IRF4 axis plays a pivotal role in regulating M2 macrophage polarization [11], suggesting that it may be a general mechanism of resistance to cell death that can be triggered in times of cellular stress.

Clinically, the expression of JMJD3 is likely to be relevant to DLBCL malignancy since it is expressed in 92% of patient samples examined. Down-regulation of JMJD3 results in amelioration of DLBCL in mouse models of disease, consistently. Although the intratumoral injection of siRNA we preformed does not seem likely to translate rapidly to the clinic, there may nonetheless be other mechanisms by which the JMJD3 axis could be targeted, including inhibitors of NF-κB, or methyl transferase inhibition in tandem with cytotoxic chemotherapeutics. Altogether, our study helps further delineate the key players in the pathogenesis of DLBCL and thus provide more desirable targets for the control of this disease.

## MATERIALS AND METHODS

### Animals and cell lines

Male NOD/SCID mice aged 6–8 weeks were purchased from the Laboratory Animal Center, the Academy of Military Medical Sciences, (Peking, China). All animal experiments were performed according to Health guidelines of Nankai University Institutional Animal Use and Care Committee. The normal B cell line HMy2. CIR was purchased from the institutes for biological sciences of Chinese academy of sciences (Shanghai, China). DLBCL lines SU-DHL-2 and SU-DHL-8 were purchased from American Type Culture Collection (Manassas, VA). Five DLBCL cell lines (OCI-Ly3, OCI-Ly19, SU-DHL-4, SU-DHL-6 and VAL) were the kind gift of Dr. Jun Cheng (Tianjin Medical University Cancer Institute and Hospital, Tianjin, China). Pyrrolidine dithiocarbamate (PDTC) was purchased from Sigma-Aldrich (#P8765, St Louis, MO).

### RNA isolation, RT-PCR and real-time PCR

Total RNA was extracted from DLBCL cells and tumor tissues from the mouse model using the TRIZOL reagent (Invitrogen). The experiment was performed according to the manufacturer's protocol. Real-time PCRs were performed using TransStart Top Green qPCR SuperMix in a CFX^™^ Real-Time Thermal cycler (Bio- Rad) for sequence detection. The primers used in RT-PCR and real-time PCR are listed in Supplementary Table S1.

### Total, cytosolic, and nuclear protein extraction

Cell lysates from human DLBCL cells or tumor tissue cells were prepared with RIPA buffer in the presence of protease inhibitor cocktails. For nuclear and cytosolic extracts, the extraction was performed according to the manufacturer's protocol of KeyGEN Nuclear-cytosol Extraction kit (KeyGEN Biotech, Nanjing, China) (Cat# KGP1100).

### Vector construction

The human IRF4 and BCL2 expression plasmids were generated by inserting IRF4 fragment between Bam H I and Xba I and BCL2 between Xba I and Nhe I sites of the multiple cloning site (MCS) of pLV-EF1α -MCS-IRES-Bsd vector (Biosettia, San Diego, CA). siRNA sequences for silencing human JMJD3 (5′-GCUACACCUUGAGCACAAATdT-3′) and IRF4 (5′-ACCUCGCA CUCUCAGUUUCdTdT-3′, 5′-UCCG AGAAGGCAUCGACAAdTdT-3′) genes were designed and chemically synthesized by RiboBio Company (Guangzhou, China). A scrambled sequence designed (RiboBio Co.) was used as control for knockdown analysis. The human JMJD3 expression vector pCMV-JMJD3-HA was purchased (Addgene, Cambridge MA).

### Protein and siRNA expression

Human DLBCL cells were transfected with pCMV-JMJD3-HA, pLV-EF1α-IRF4-IRES-Bsd, pLV-EF1α-BCL2-IRES-Bsd expression plasmids alone or co-transfected with siRNA of JMJD3. 10^6^ DLBCL cells were plated in the 12- well plate with medium without penicillin-streptomycin, then transfected with Lipofectamine^™^ Transfection Reagent (Invitrogen) as per manufacturer's instructions. Protein levels were determined by immunoblot. The effect of JMJD3 overexpression or suppression was investigated in cells either harvested for immunoblotting, RT-PCR and real-time PCR, or used for proliferation, apoptosis and CHIP assays after 48 hours of transfection.

### Immunoblot and immunohistochemical analysis

Detection of protein expression by immunoblot was carried out according to the established protocols described previously [40]. JMJD3 (Cell Signal, Danvers, MA) (Cat# 3457), IRF4 (Abcam, Cambridge, MA) (Cat# ab5184- 1), caspase-3 (Cell Signal) (Cat# 9665), Bcl-2 (Cell Signaling), (Cat# 2870), NF-κB (P65 subunit) (Abcam) (Cat# ab32536), NF-κB (P105/50 subunit) (Abcam) (Cat# ab32360), laminA (Cat# ab8980), (Abcam), IκBα (1:1000, Cat# ab32518, Abcam), p-IκBα (1:1000, Cat# ab133462, Abcam) and β-actin (Santa Cruz Biotechnology, Inc., Santa Cruz, CA) (Cat# sc-47778) antibodies were used. Secondary horseradish peroxidase - conjugated goat anti-rabbit or mouse antibodies (Bio-Rad) were detected by the enhanced chemiluminescence reagent (Millipore, Billerica, MA). Paraffin sections were incubated at 4°C overnight with the primary antibody after dewaxing and hydration. Anti-JMJD3 (Abcam) (Cat# ab38113) or -IRF4 (Abcam) were used at 1:50 dilution. Slides were incubated with a biotinylated secondary antibody for 1.5 h, then developed with avidin-peroxidase and DAB and counterstained with hematoxylin. Dehydrated slides were mounted with neutral resin.

### Analysis of apoptosis and proliferation

For annexin-V/PI analysis, cells were stained according to the manufacturer's instructions (BD Biosciences, San Jose, CA) and cells positive for annexin-V detection were measured using FlowJo software (BD Biosciences). Proliferation assays were performed as per manufacturer's protocol (Cell Proliferation Assay, Cell Counting Kit-8 (CCK- 8, Dojindo Laboratories, Shanghai, China) after seeding 2000 cells in 96- well plates in complete medium 24 h following transfection with control vector, pCMV-JMJD3-HA vector or JMJD3 or scrambled siRNA.

### Chromatin immunoprecipitation (ChIP)

OCI-Ly3 cells in T75 flask were cross-linked by 1% formaldehyde. Glycine was used to quench the unreacted formaldehyde. The cells were harvested and sonicated following the instruction of EZ-ChIP™ kit (Millipore) (Cat# 17–371). Sample was centrifuged and supernatant was collected and first precleared with Protein G Agarose, then 10 μl supernatant was saved as input control. 1 μg anti-RNA Polymerase, 1 μg Normal Mouse IgG or 5 μg Mouse anti-Histone H3 (trimethyl K27) antibody (Abcam) (Cat# ab6002), was added to each supernatant fraction in tube used as the positive control, negative control, or H3K27me3 sample respectively and rotated overnight at 4°C. The DNA purified from both input control samples and immunoprecipitations were subjected to PCR amplification for a 300 bp fragment of the IRF4 promoter. All PCR signals from immunoprecipitated DNA were normalized to PCR signals from non-immunoprecipitated input DNA. The primers used in PCR are listed in Supplementary Table S2.

### Therapeutic treatment

Mice were separated randomly into two groups (*n* = 10 for each group). 1 × 10^7^ OCI-Ly3 (ABC subtype) or OCI-Ly19 (GCB subtype) cells were xenografted into each mouse through subcutaneous injections. 32 days later, mice received 4 times of tumor multipoint injections, at 3-day intervals, of control siRNA or JMJD3 siRNA (10 nmol per mouse). Mice were sacrificed 24 hours after the final treatment and body weights and tumor volumes were determined. The tumor tissues subjected to further analysis. TUNEL (Promega, Madison, Wisconsin) immunohistochemical staining was performed according to the manufacturer's protocol.

### Statistical analysis

All data are presented as mean ± S.E.M. Statistical analysis of the data was performed using the GraphPad Prism software (GraphPad Soft ware, Inc. La Jolla, CA). Differences between individual groups were analyzed by paired *t*-test or *χ*^2^, as appropriate. *P* value < 0.05 was considered statistically significant.

## SUPPLEMENTARY MATERIAL FIGURES AND TABLES


