# Supplementary material for: JMJD3 promotes survival of diffuse large B-cell lymphoma subtypes via distinct mechanisms
Source: Oncotarget. 2016 Apr 19;7(20):29387–99. doi: 10.18632/oncotarget.8836 (PMC5045403; doi:10.18632/oncotarget.8836)
Supplement: Supplementary file 1 [file oncotarget-07-29387-s001.pdf]

JMJD3 promotes survival of diffuse large B-cell lymphoma subtypes via distinct mechanisms

Supplementary Materials

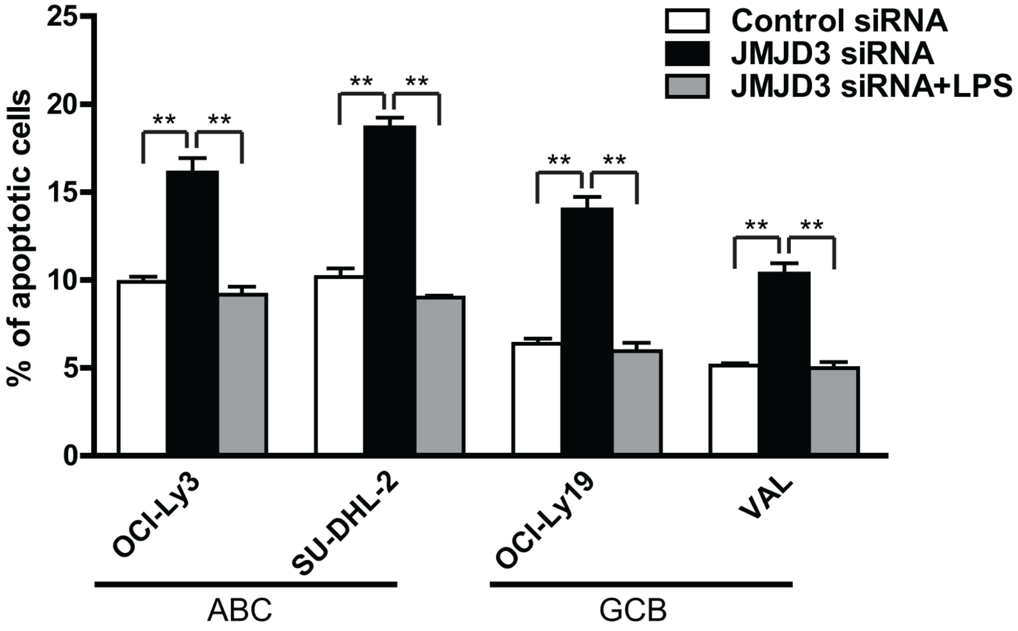

Supplementary Figure S1: NF-κB activation induced by Lipopolysaccharides (LPS) rescue the pro-apoptotic effect of JMJD3 downregulation in DLBCL cells. Human DLBCL cells were transfected with JMJD3 or scramble control siRNA for 24 hours, then treated with or without LPS (1 µg/ml) for another 24 hours. Apoptosis was detected by FACS assay using annexin-v/PI staining. Data shown are the mean ± S.E.M. of three independent experiments (**$P < 0.01$, student's t-test).

Supplementary Table S1: Primers for polymerase chain reaction (PCR) assay

Primer name	Primer sequences (5′ → 3′)	
	Sense	Antisense
Homo IRF1	CCAGAGCAGGAACAAGGG	TGTGGTCATCAGGCAGAG
Homo IRF2	TTCAGGGTCTACCGAATGCT	TGATGTTCACCGTACTATCCACT
Homo IRF3	CAGCAGGAGGATTTCGG	CCCTTCTTTGCGGTTGAG
Homo IRF4	CAACGCCTTACCCTTCG	GGCTTCGGCAGACCTTAT
Homo IRF5	CCAAGGAGACAGGGAAAT	CGTAGATGAGGCGGAAG
Homo IRF6	GGCTGCCGACTCTTCTATG	CCTGGGAATTTGACCTGCT
Homo IRF7	CAGCAGGTAGCATTCCC	CAGCAGTTCCTCCGTGT
Homo IRF8	ACATTTCCGAGCCATACAA	AGCGACCGCACTCCATC
Homo IRF9	CAGGATGCTGCCTTCTTC	TGTGCTGTCGCTTTGATG
Homo Bcl-2	ATCCAGGATAACGGAGGCT	CAGGAGAAATCAAACAGAGGC
Homo JMJD3	CCACAGCGCCCTTCGATAC	CCATAGTTCCGTTTGTGCTCAAG
Homo Pax5	CTTGGCAGGTATTATGAGACAG	GGCAAACATGGTGGGATT
Homo Blimp1	TAAGAACGCCAACAGGAAATA	CTGCAAAGTCCCGACAATAC
Homo GAPDH	CTGATGCCCCCATGTTCGTC	CACCCTGTTGCTGTAGCCAAATTC

Supplementary Table S2: Primers used for ChIP assay

Primer name	Primer sequences (5′ → 3′)	
	Sense	Antisense
Chip-IRF4-1	CTAAAAGCAGAGGATGTGGA	TTTCTTCCTTTCCTTTCACA
Chip-IRF4-2	ACAAGTAGCAGGTGCTCAAA	GCATTTACTTCGCATTTTCA
Chip-IRF4-3	CCTGAAAATGCGAAGTAAAT	TGTGGCTCAAGCCTGTAATC
Chip-IRF4-4	AAAGTGCTGGGATTACAGGC	GAGAACATCGCACTCACTAAA
Chip-IRF4-5	AGCATGTCAGACACGCAGAG	TCAGGAGGCCAGTCAATCA
Chip-IRF4-6	TGAGGTCCTGGCGCAAAGG	CCCAAGATCGAGCGGTGAAA
Chip-BCL2-1	TGTGGGAGCAAAGGAAGAC	CTGATGGTTGGGACAGAGT
Chip-BCL2-2	GACTCTGTCCCAACCATCA	CCCTGCCTGACATCTTTAT
Chip-BCL2-3	GGCACAGTGGCTCATGTCT	AGCGGCTTACTTAATAGGG
Chip-BCL2-4	CCCAATAATCCAGTGTCCC	GTCTCCACCTTTGCCTCGT
Chip-BCL2-5	CTTTAGGAGCCCACCCAC	CTGCCCTGCTGTGAAGAC
Chip-BCL2-6	GGCATTGGCCTGGGTCT	CGTGCGGACTTGGTGGT
Chip-BCL2-7	CCCCAGCGACCACCAAGT	GGCTGTGGTGCCTGTCCTCTT
